# Comparison of transcranial brain stimulation approaches: prefrontal theta alternating current stimulation enhances working memory performance

**DOI:** 10.3389/fpsyt.2023.1140361

**Published:** 2023-06-30

**Authors:** Jonas Rauh, Anne S. M. Müller, Guido Nolte, Moritz Haaf, Marius Mußmann, Saskia Steinmann, Christoph Mulert, Gregor Leicht

**Affiliations:** ^1^Psychiatry Neuroimaging Branch, Department of Psychiatry and Psychotherapy, University Medical Center Hamburg-Eppendorf, Hamburg, Germany; ^2^Department of Neurophysiology and Pathophysiology, University Medical Center Hamburg-Eppendorf, Hamburg, Germany; ^3^Center of Psychiatry, Justus-Liebig University, Giessen, Germany

**Keywords:** working memory, transcranial alternating current stimulation, transcranial direct current stimulation, theta oscillations, dorsolateral prefrontal cortex, high-definition transcranial electrical stimulation

## Abstract

**Introduction:**

One of the most important cognitive functions in our everyday life is the working memory (WM). In several neuropsychiatric diseases such as ADHD or schizophrenia WM deficits can be observed, making it an attractive target for non-invasive brain stimulation methods like transcranial electrical stimulation (tES). However, the literature shows rather heterogeneous results of tES effects on WM performance. fMRI meta-analyses have identified a WM network including frontoparietal brain areas such as the dorsolateral prefrontal cortex (DLPFC) and the posterior parietal cortex (PPC). Neurophysiological studies revealed oscillatory activity in the theta band frequency range to be of crucial functional relevance for WM processes. Based on this, transcranial alternating current stimulation (tACS) in the theta frequency range targeting DLPFC and PPC in a spatially optimized way might further improve effects of tES on WM performance.

**Methods:**

Sixteen healthy subjects were stimulated with varying stimulation settings on four different days in a counterbalanced within-subject design. These setups included the application of (1) tACS with a frequency of 5 Hz (theta frequency range) over the left DLPFC and (2) the right superior parietal cortex, (3) transcranial direct current stimulation (tDCS) of the DLPFC and (4) a sham stimulation condition during the online performance of a visual delayed-match-to-sample task with varying working memory load. We introduce a procedure to calculate an optimal tES model revealing optimized high-density setups for the present study for 3 cathodes and 1 anode and stimulation currents of 1.5 mA.

**Results:**

A significant interaction effect of stimulation type and load condition on working memory capacity was found. This was reflected by a significant improvement of WM performance in the high load condition during tACS over the left DLPFC compared with sham stimulation, which was not the case for our parietal tACS or tDCS setup.

**Discussion:**

Working memory performance can be improved with optimized high-definition tACS with a frequency of 5 Hz over the left DLPFC. The conception of different mechanisms underlying transcranial electrical stimulation with alternating and direct currents is supported by these results. Patients suffering from working memory impairments due to neuropsychiatric diseases might potentially benefit from this brain stimulation approach.

## 1. Introduction

Within the last decade, there has been growing interest in neuroscience on the potential benefit of non-invasive brain stimulation techniques such as transcranial electrical stimulation (tES) during cognitive tasks (1).

One component tES has been focusing on is the working memory (WM), which is involved in nearly all cognitive functions in our everyday life including language, problem solving, and abstract thinking (2). Deficits of the WM are observed, for example, in neuropsychiatric diseases like schizophrenia (3, 4), ADHD (5), or Alzheimer’s disease (6, 7) and play a major role in the severity of the illnesses.

A vast number of functional magnetic resonance imaging studies and meta-analyses have proven the involvement of various brain regions including primarily the prefrontal cortex, superior and inferior parietal lobules, and the inferior temporal cortex during visuospatial WM paradigms (8, 9). While prefrontal activity was initially assigned to maintained information, more recent studies indicate a rather organizing and controlling role of the prefrontal cortex, guiding for example to representations of sensory stimuli in more posterior regions (10–12).

A positive impact of anodal transcranial direct current stimulation (tDCS) was first reported by Fregni and colleagues (13). Several studies have replicated enhancement effects of anodal tDCS on WM performance. Most of them reported improved accuracy (14–16) (for review *cf.* (17)), while others showed shortened reaction times (18, 19). In accordance with fMRI studies, the location of the anodal stimulation was predominantly chosen over the left dorsolateral prefrontal cortex (DLPFC) at electrode F3 according to the 10–20 system used for electrode positioning during EEG measurements (17, 20).

Several animal experiments suggest that the main effect of tDCS relies on a subthreshold modulation of resting membrane potentials of the neurons. Specifically, cortical excitability located within the electric field underneath the stimulation electrode can be increased with anodal stimulation and decreased with cathodal stimulation (21, 22). In contrary, during tACS excitability of the neurons is modulated by an alternating anodal and cathodal stimulation in a specific rhythm through a continuous alternating resting membrane potential shift. Depending on the chosen frequency and strength of the electric field, this can lead to an neural entrainment of endogenous oscillations (23–27) and network resonance (28). Neuronal oscillations appear to be an important mechanism for coupling of brain areas (29). Many EEG studies have emphasized the importance of oscillations in the theta frequency range (4–8 Hz) for different cognitive functions such as memory processes (30) or spatial navigation (31, 32). Functional dominance of theta activity can be observed during WM tasks (33, 34). Frontal midline theta not only plays an important role regarding cognitive control (35), but particularly is essential for successful manipulation of the WM (36, 37). Some studies have shown a variation of theta activity related to alterations of the WM load (38, 39). Simultaneous EEG-fMRI measurements (40) and intracranial recordings (33, 41) support the assumption of (pre-)frontal and parietal cortices as generators of theta oscillations during WM performance.

In light of these findings and of a study reporting that temporal firing patterns can be influenced depending on the stimulation frequency applied via sinusoidal electrical fields (23) the assumption can be made, that WM functioning could also be improved by transcranial alternating current stimulation (tACS) through entrainment of theta power. Based on this, several studies to date have investigated the influence of tACS within the theta range on performance and electrophysiological parameters, mainly stimulating the parietal cortex or the frontal cortex or both (42–54). Positive effects on working memory performance following parietal stimulation is often reported during stimulation of the right side (48, 53, 55). However, nearly all these studies employed conventional setups with single electrodes over target brain regions and some (43, 44, 49, 52) focused on effects after stimulation, e.g., based on spike-timing dependent plasticity (56, 57).

While classical transcranial electrical current setups used two large rectangular stimulation electrodes (i.e., 35 cm^2^) and the applied current therefore spreads widely throughout the brain, subsequent studies have proposed more focal stimulation setups. In these setups an anodal stimulation electrode is surrounded by several cathodal electrodes (58, 59), which results in a confined electrical stimulation field within the electrode ring. Research regarding such high-definition (HD)-tACS is a novel area, and there is a lack of studies on the practical impact of this method on visual WM. For the first time, we present a procedure to create an optimized stimulation model using HD stimulation in this paper.

Our main hypothesis is that HD-tACS of brain regions involved during WM tasks, namely the DLPFC (middle frontal gyrus, BA9/BA46) or the superior parietal lobule (BA40), can enhance WM performance measured by means of WM accuracy, capacity, or reaction times compared with a sham stimulation condition. Assuming that oscillatory activity is an important feature for the interaction of different brain regions within functional networks, we also expect a superior improvement through HD-tACS compared with HD-tDCS. Further, we assume that there are differences regarding the different stimulation sites (i.e., frontal vs. parietal).

## 2. Methods

### 2.1. Ethics statement

The present study was part of a larger project investigating the modulation of disturbed networks in schizophrenia with transcranial electrical stimulation, within the context of the Collaborative Research Centre 936 (“multi-site communication in the brain,” www.sfb936.net). The study was approved by the Ethics Committee of the Medical Association Hamburg and carried out in accordance with the latest version of the Declaration of Helsinki. Written informed consent was obtained from all participants after the aim of the study and the nature of the procedures had been fully explained.

### 2.2. Participants

16 healthy participants [6 male, all right-handed, mean age 34.8 years (SD 11.7)] were included in the study. To estimate the required sample size, we had conducted a power analysis for a repeated-measures ANOVA with 8 measurements, alpha level of 0.05, power of 0.8 and a moderate effect size (*f* = 0.25). The estimated sample size based on these assumptions was *n* = 16. Exclusion criteria for all participants were any previous psychiatric disorder or treatment, a family history of psychotic disorders, current substance abuse or dependence, and presence of major somatic or neurological disorders.

### 2.3. Study design

Each participants obtained four separate sessions of measurements of performance in a working memory task as described below. During two of these sessions HD-tACS was applied simultaneously with WM task performance over frontal or parietal regions, respectively. Frontal HD-tDCS or a sham stimulation were conducted during the task in the other two sessions. The sessions were at least 3 days apart from each other. The order of the sessions was pseudo-randomized and balanced between subjects. The subjects were blinded with respect to the kind of stimulation applied (pseudo-randomized single-blinded cross-over design). On each day participants filled out a questionnaire asking for adverse effect of the stimulation (itching, burning, heating, metallic taste, fatigue, other) and whether they believed that they received a stimulation or not or they did not know.

### 2.4. Paradigm

A visual WM delayed matched to sample reaction task based on a work from Haenschel and colleagues (60) was used as paradigm containing the presentation of non-natural visual objects (blurred outlines of random tetris shapes = BORTs) in two conditions with varying WM load. BORTs have the advantage of being novel and difficult to verbalize. We used a larger number of different BORTs objects to avoid lasting associations through recognition of previously viewed stimuli and subsequent ceiling effects. For this purpose, 504 (448 for actual session, 56 for training) single visual objects were built with a custom-written MATLAB script. Within one session no stimulus recurred except for matching probe stimuli.

**Figure 1:**
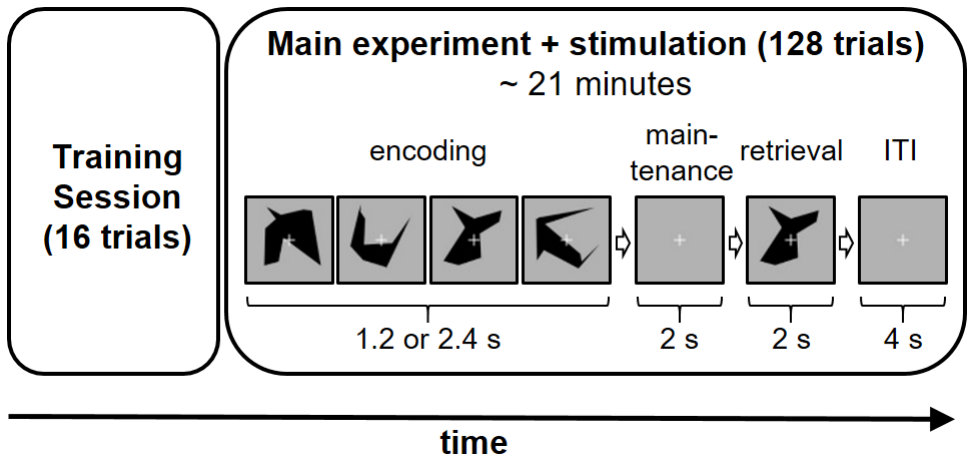
Experiment sequence on each study including example trial sequence (high load condition) day. Applied stimulation method (frontal HD-tACS, parietal HD-tACS, frontal HD-tDCS or sham stimulation) was varied on each of the 4  days in a counterbalanced sequence.

The Presentation software (Neurobehavioral Systems, Berkeley, United States) was used for stimulus presentation. One trial consisted of an encoding, a maintenance, and a retrieval phase (Figure 1). During the encoding phase two (low load condition) or four (high load condition) different visual objects were shown for 600 ms in a row resulting in a duration of the encoding phase of 1.2 to 2.4 s depending on the different condition. After the encoding phase a fixation cross was shown for 2 s. The participant was instructed to memorize the displayed items during this maintenance phase. In the following retrieval phase a probe stimulus was shown for 2 s and the participant was asked to indicate as fast and accurately as possible whether this probe stimulus had been shown during the encoding phase via button-press with the left (mismatch) or the right (match) index finger. The intertrial interval was set to 3.5 s.

The position of the target stimulus (respectively the first, second, third or fourth stimulus of the encoding phase) was equally distributed and did not change for one set of a trial.

One session of the experiment consisted of 128 trials (64 per each condition) which were presented in a randomized order. A training session of 16 trials was conducted at the beginning of each experiment day.

### 2.5. Stimulation models

#### 2.5.1. Calculation of an optimized transcranial electrical stimulation model

Following our hypotheses, we needed two different montages of electrodes to stimulate over the left dorsolateral prefrontal cortex or the right parietal cortex. To this end, we built our high-definition stimulation models using an optimization algorithm here described as the procedure to calculate an optimal tES model at a given location and orientation inside the head for N stimulation electrodes. The optimization is done both with respect to the amount of inserted current at the stimulation electrodes and to the position of these electrodes. We first describe the optimization for given electrode locations and then the optimization with respect to the locations. These two steps are combined for the full optimization.

The electric field induced by inserting a unit current at some location (the stimulation electrode) and extracting it some other location (the reference) is well known to be equal to the electric lead field representing the sensitivity of an EEG sensor at that location (with that reference) to source activities. Hence, calculating transcranial electrical stimulation models is equivalent to solving the EEG forward problem, for which an analytic expansion of the electric lead field in spherical harmonics for a three-shell volume conductor of realistic shape was used (61). Within the volume inside the innermost shell a grid with neighboring grid-points having 5 mm distance is defined, and EEG forward solutions are calculated for a fixed set of M electrodes with M> > N. To optimize with respect to N electrodes, a subset of the full lead field will be used.

We denote by L(i,j,k) the electric potential at the *i*th sensor (with i=1…N) for the *j*th grid-point, induced by a source of unit magnitude pointing into *k*th direction with k=1,…,3. We recall that the lead field *L* is the reduced version of the full lead field, and it is transformed to average reference, i.e., for each *j* and *k* we subtract the average of *L* across the *N* sensors. The electric field induced by a tES of magnitude α(i) for the *i*th sensor induces, apart from an irrelevant constant, an electric field at the *j*th grid-point in the *k^th^* direction equal to


$$E\left(j,k\right)=\underset{i}{\sum }L\left(i,j,k\right)\alpha \left(i\right)$$

The goal is to optimize the stimulation *α(i)* such that the induced electric field is fixed in a specified direction a specified grid-point inside the head while simultaneously minimizing the stimulation of all other regions and directions. The latter can be defined in various ways, and we here chose to minimize the (square of the) 2-norm of the electric field leading to minimize the cost function


$$H=\underset{jk}{\sum }E^{2}\left(j,k\right)$$

under the constraint


$$\underset{i}{\sum }L_{0}\left(i\right)\alpha \left(i\right)=1$$

where $L_{0}\left(i\right)$ is the topography of an electric dipole at the specified location with specified orientation. It is possible (but not necessary) to choose the location at one of the grid-points, say the *m*^th^. If the source direction is given by u(k) with k=1,..,3 then


$$L_{0}\left(i\right)=\underset{k}{\sum }L\left(i,m,k\right)u\left(k\right)$$

Minimizing the cost function H under the constraint can be solved analytically and results in


$$\vec{\alpha }=\frac{1}{\lambda }K^{-1}{\vec{L}}_{0}$$

with


$$K\left(p,q\right)=\underset{j,k}{\sum }L\left(p,j,k\right)L\left(q,j,k\right)$$

and


$$\lambda ={\vec{L}}_{0}^{T}K^{-1}{\vec{L}}_{0}$$

The above formulation is only valid if *K* is invertible which is not the case if the reference electrode is included in the lead field tensor *L* or if, e.g., the topographies are referenced to common average reference as was done here. In such a case the inverse of *K* can be replaced by its pseudo-inverse. A more general approach is to regularize *K* and make the replacement


K→K+βid

where *id* is the identity matrix in the N-dimensional sensor space. Replacing the inverse of *K* with its pseudo-inverse corresponds to the limit *β *→*0*, while choosing a finite value for *β* leads to less total current inserted to the head compromising on the cost function. In this paper we have chosen a rather small regularization of


$$\beta =10^{-4}\frac{tr\left(K\right)}{N}$$

Since we used a lead field with average reference, the total inserted current will vanish as it should.

Using this solution for given electrode positions we optimize across positions by a simple random search. We start with a random subset of N electrodes out of the total of M electrodes and calculate the cost function H for that configuration. Then we replace a random electrode from the N electrodes by a randomly chosen other electrode from the total of M electrodes and recalculate H. If H is now lower than, this change is accepted as an improved configuration. This is repeated 2000 times and for 10 different initial conditions. We found that in more than 70% of the initial conditions the solution converges to the same configuration with an absolute minimum of the cost function across all tested initial conditions.

#### 2.5.2. Stimulation parameter

In total, 4 different stimulation protocols were applied on 4 different days for each subject. These models contained a “left frontal” HD-tDCS, a “left frontal” HD-tACS, a “right parietal” HD-tACS and a sham stimulation.

For the electrical stimulation, a DC Stimulator MC (neuroConn GmbH, Ilmenau, Germany) was used. The stimulation types covered two alternating currents with a stimulation frequency of 5 Hz for both montages, a direct current stimulation at the left frontal montage and a sham stimulation at the right parietal montage. We decided to use a stimulation frequency of 5 Hz, as the medium frequency of the theta range (3–7 Hz). As theoretically suggested by the principle of the Arnold tongue (62), using the medium frequency of a frequency range suspected to be involved in a cortical process for stimulation more likely results in entrainment effects in a group analysis, because in this case the stimulation frequency has a higher probability to be close to the target frequency of individual subjects. Moreover, 5 Hz activity as an approximation for theta activity has been used in previous work on EEG patterns related to working memory processes during a visual delayed match to sample task similar to the one used in our study (60). For the sham condition the stimulation duration was 10 s ramp in, 10 s stimulation and 30 s ramp out, while all other conditions lasted 21 min in total (including 10 s ramp in and 10 s ramp out).

Each montage consisted of one “anodal” rubber electrode with a diameter of 2 cm that was surrounded by 3 “cathodal” Ag/AgCl electrodes. An EEG cap was placed initially, and the target position of the rubber electrode was marked by a pen. The cap was then removed and the rubber electrode was attached to the head via Ten20 paste (Weaver and Company, Aurora, USA). Then the EEG cap was again fitted to the head. The remaining stimulation electrodes were directly integrated within the cap. To minimize their impedances Signagel® Electrode Gel (Parker Laboratories, Fairfield, USA) was used.

**Figure 2:**
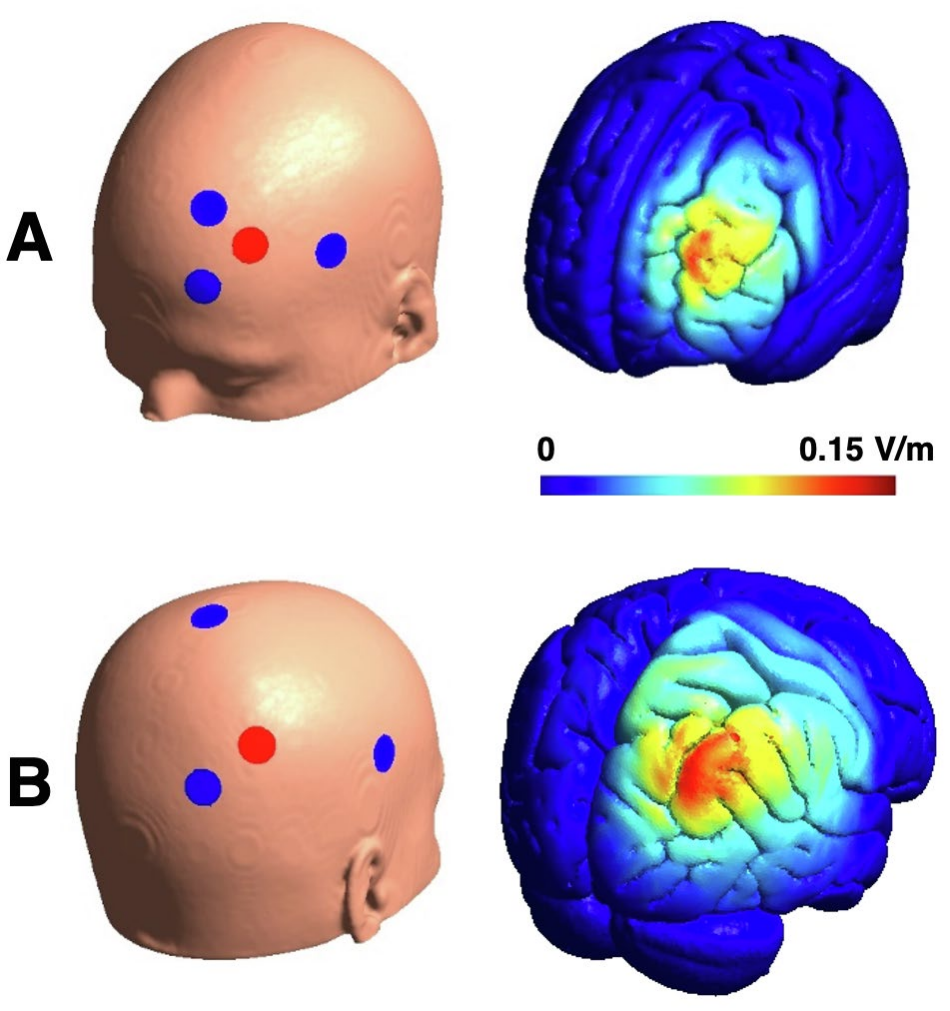
Electrode montages and their electric field distributions for amplitude peaks of tACS. Panel **(A)** displays example for frontal HD-tACS. HD-tDCS shows the same distribution with higher electric field intensities (E_max_ = 0.27 V/m). Change of the electric field within one tACS cycle is comparable to tDCS (2 × E_max_ = 0.137 V/m). Panel **(B)** displays the electrode montages for parietal HD-tACS and sham stimulation and the electric field for the parietal stimulation (E_max_ = 0.133 V/m).

According to reports on effects on left prefrontal and right parietal transcranial electrical stimulation on working memory performance (17, 48, 53, 55), we targeted these regions with different stimulation approaches. Targeting the left dorsolateral prefrontal cortex, we chose a target position of (*x, y, z* = −40, 37, 24) in the MNI space (based on the search term “dorsolateral prefrontal” on neurosynth.org). As a result, we calculated a “left frontal” high-definition model consisting of 3 cathodes at the positions F1, FC5 and AF3 and one anodal electrode at F3. To obtain a stimulation current of 1.5 mA (peak-to-peak for tACS) at F3, the stimulation currents were weighted −690 μA at F1 and − 405 μA at FC5 and AF3, respectively. For the other montage targeting the right parietal cortex (right parietal, MNI: *x, y, z* = 41, −42, 47, search term: “parietal”), we again calculated a high-definition model consisting of 3 cathodes with the positions CPz, PO4, and C6 and one anode at P4. To obtain a stimulation current of 1.5 mA (peak-to-peak) at P4, the currents were set to −840 μA at PO4 and 330 μA at C6 and CPz, respectively. Electric field simulations were calculated using SimNIBS (63) (Figure 2).

### 2.6. Parametrization and statistical analysis

For statistical analysis of the data SPSS (Version 29.0, IBM) was used. Error rates, reaction times and working memory capacity were calculated for every subject, stimulation day and WM load. Error rates were defined as the number of incorrect answers divided by the number of trials. Reaction times were defined as the average timespan between probe stimulus and correct answer, while the working memory capacity (WMC) was determined by Pashler’s formula (64). This formula was commonly applied in other WM paradigms (60, 65) and reads


$$WMC=\frac{n\left(h-g\right)}{g}$$

where *n* is the number of presented items (2 or 4), *h* is the hit rate (number of correct matches), and *g* the false rate (number of wrong non-matches). A 4 × 2 repeated-measures ANOVA including *condition* (tACS parietal, tACS frontal, tDCS frontal and sham) and *load* (low load/2 items and high load/4 items) as factors were calculated. To account for possible gender effects, we added gender as a between subject factor in the ANOVAs. Post-hoc tests were corrected for multiple testing using the Bonferroni correction. Additionally, ANOVAs with *condition* as one factor were calculated for every reported adverse effect (*cf.* section 2.3). Success of blinding was assessed by a chi-square-test.

## 3. Results

### 3.1. Blinding and adverse effects

Participants could not differentiate, if the sham stimulation was a real stimulation or a sham stimulation as shown by a chi-square-test (*χ*^2^ (3) = 3.875, *p* = 0.144). Altogether, stimulation was very well tolerated as the mean of reported adverse effects was below *light*, except for fatigue. Average fatigue was between light and moderate, but in all conditions including sham stimulation. Fatigue can be observed in any psychological paradigm and is probably not directly linked to the stimulation. Furthermore, no ANOVA of the single adverse effects showed any significant stimulation effect.

**Figure 3:**
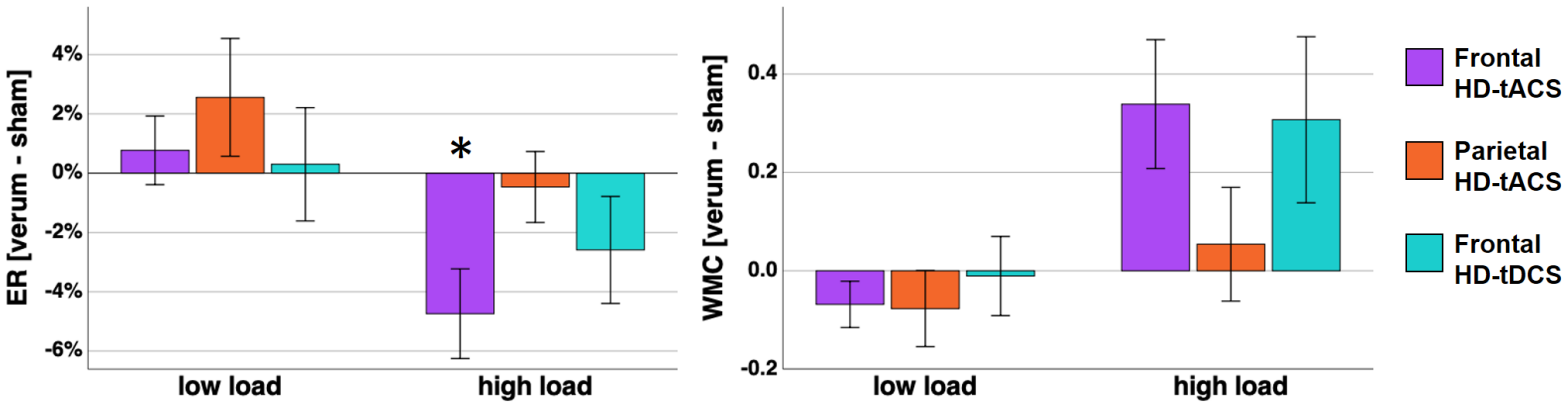
On the left panel mean error rates differences between verum stimulation (frontal HD-tACS, parietal HD-tACS, frontal HD-tDCS) and sham stimulation are displayed. The changes of WMC are displayed correspondingly on the right panel. Error bars represent standard error of the mean. Asterisk indicates a Bonferroni-corrected value of *p* <0.05.

### 3.2. Error rates

In the two-factor repeated measures ANOVA a main effect of *load* was found (F (1, 15) = 104.843, *p* < 0.001, η^2^_p_ = 0.875). Additionally, an in-trend effect of *stimulation x load* was found (F (3, 45) = 3.209, *p* = 0.072, η^2^_p_ = 0.143). However, error rates in the rather easy low load condition were confounded by ceiling effects (i.e., error rates converging toward 0). Thus, we calculated an explorative follow-up ANOVA for high load condition only with one factor *stimulation*. For the high load condition ANOVA we found a significant effect of stimulation (F (3, 45) = 3.449, *p* = 0.024, η^2^_p_ = 0.187). Post-hoc tests indicated significant lower error rates during frontal HD-tACS compared with sham stimulation (T (15) = −3.134, *p* = 0.041, d = −0.783, Figure 3). No significant effect of gender was found in the ANOVA with either load factor included (*p* = 0.535) or the ANOVA for high load condition error rates only (*p* = 0.996). To further explore the development of the stimulation effect over time, we calculated a 2 × 4 ANOVA including the factors *stimulation* with the steps frontal tACS and sham stimulation and *time* with 4 time points reflecting the error rates of 16 sequential trials of the high load condition. However, we did not find a significant interaction (*p* = 0.606), nor a main effect of time (*p* = 0.204).

### 3.3. Working memory capacity

In the two-factor repeated measures ANOVA a *load* effect (F (1, 15) = 13.270, *p* = 0.002, η^2^_p_ = 0.469) and a *stimulation x load* interaction (F (3, 45) = 3.418, *p* < 0.025, η^2^_p_ = 0.186) were found. Subsequent post-hoc pairwise comparisons including multiple comparison correction did not reveal any significant differences between the conditions. However, at least without the correction, a higher WMC was found in the high load condition during frontal HD-tACS compared with the sham stimulation (T (15) = 2.586, *p* = 0.041, d = 0.646). When adding gender as a between subject factor, this resulted in no significant effect of gender (*p* = 0.915).

### 3.4. Reaction times

For reaction times a significant effect for *load* was found in the repeated measures ANOVA (*F* = 14.871, *p* = 0.002, η^2^_p_ = 0.498). Neither the *stimulation* effect (*p* = 0.790) nor the interaction (*p* = 0.742) did reach significance for reaction times.

## 4. Discussion

The main goal of our study was to investigate if different stimulation types and targets show comparable or different effects on the working memory performance. The present study measured and compared WM performance alterations during an HD-tACS theta stimulation over the DLPFC, an HD-tACS over the parietal cortex, a HD-tDCS over the DLPFC and during a sham stimulation. For our HD stimulation approach, we introduce and use for the first time a new procedure to calculate an optimal tES model at a given location and orientation inside the head for *N* stimulation electrodes for the positioning of the stimulation electrodes and weighting of the stimulation currents. Only optimized HD-tACS theta stimulation over the DLPFC led to a significant increase of the working memory capacity and accuracy during the high load condition.

### 4.1. Frontal theta-tACS

In line with our results, there have been some studies that have shown positive impacts of online frontal (42, 46, 66) or fronto-parietal theta-tACS (52, 67) on paradigms involving working memory processes, but only two studies (46, 67) applied a focal stimulation with a HD positioning of the electrodes. Some studies also report failure to produce positive effects using tACS on working memory (54, 68, 69). One possible explanation for negative results could be that conventional stimulation setups often lead to undesired stimulation of regions next to the target area which may interfere with the effects on behavioral outcomes. This might be especially relevant for stimulation of frontal areas where the return electrode often is positioned for example supraorbital. We found no interaction effect between stimulation and time. This finding might indicate that stimulation tends to have an effect on WM performance already early after start of the stimulation that is maintained over time.

### 4.2. Frontal tDCS

In line with a recent meta-analysis investigating enhancement of the working memory performance using HD-tACS (70), we did not find a significant effect on working memory performance using our optimized setup. Although there are meta-analyses propagating positive effects of tDCS on WM (20, 71), newer meta-analyses question these results (17, 72, 73). In a meta-analysis using electric field modeling Wischnewski et al. (74) found most tDCS effects on working memory performance explained by stimulation of the lower dorsolateral prefrontal cortex, which is in line with the target region we chose in our experiment. Several meta-analyses found evidence for a positive impact of frontal tDCS on WM performance after applying tDCS during multiple WM training sessions beforehand (17, 70, 75). Although we did not find a significant effect on WM performance during tDCS, there was nominally a positive effect. The low effect size might be more pronounced in a larger sample. Perhaps this is a hint that our optimized stimulation model would also be well suited for applying it during multiple WM training sessions in order to improve WM performance in general.

### 4.3. Comparison of stimulation protocols

Although only frontal HD-tACS showed a significant effect on working memory performance, we found no significant differences regarding WM performance between the frontal HD-tACS and the frontal HD-tDCS conditions. Therefore, we cannot conclude a general advantage of HD-tACS over HD-tDCS from our results. Still, the results are an indication that frontal HD-tACS is better suited for improving the working memory performance. To the best of our knowledge, only a handful of studies have directly compared the effects of tDCS and theta-tACS (47, 51, 54) or gamma-tACS (68, 76) on working memory, yet. In line with our results, in two of these studies (47, 51) tACS protocols showed better outcomes compared to tDCS protocols. These findings are further supported by a comprehensive systematic review comparing the effectiveness of studies utilizing tDCS or tACS in improving working memory performance (73). The authors of the review found moderate effects of single-session tACS or multi-session tDCS, but not for single-session tDCS. Studies investigating associative memory processes also report on advantages of tACS compared with tDCS (77, 78), while others (79) found positive effects for both stimulation types.

Reduction of the error rates were noted only during tACS of the prefrontal cortex, not during tACS of the parietal cortex. This reinforces that our finding regarding the optimized theta HD-tACS is specific for the prefrontal cortex and depends on the stimulated location. Thus, our results underline the importance of the DLPFC for WM processes and confirm this region as a well-suited target for non-invasive brain stimulation for the future, for instance also in clinical studies.

### 4.4. Parietal theta-tACS

For our optimized parietal theta HD-tACS we chose the superior parietal cortex as a target region. The calculation of our stimulation model resulted in P4 according to the 10–20 system as an optimal position for the anodal electrode. The same electrode position was used in earlier studies successfully improving working memory performance (43, 48, 53). Contrary to our hypothesis, our model did not have an impact on neither accuracy nor capacity nor reaction times during performance of the WM task. In summary with the results regarding our frontal HD-tACS setup this contradicts the findings of other studies (43, 44) in which a parietal stimulation was favorable regarding an improvement of WM performance compared to a frontal stimulation. However, in both studies conventional, but not HD-tACS was used and offline effects were examined. The importance of theta frequency for the success of parietal tACS also appears noteworthy. In line with this, there is evidence that effects of parietal tACS on WM performance are higher for stimulation frequencies in the lower theta range (i.e., 4 Hz) compared with higher theta frequencies (i.e., 7 Hz) (45, 47, 48, 53, 55) or at least stimulation frequencies close to the individual endogenous frequency involved in WM processes (69). This follows the cross-frequency coupling theory from Lisman et al. (80) which postulates that each of the several gamma subcycles within a theta cycle represents an item to be encoded. If then the theta frequency is lowered, e.g., through entrainment following tACS more items might be stored. Since we have chosen a fixed frequency of 5 Hz and do not have information about the individual endogenous theta frequency, we are not able to evaluate this mechanism in our study.

### 4.5. Limitations

Several limitations of this study need to be considered. The number of subjects is rather low, however, unlike most other studies investigating tES we have used a within-subject design, which accounts for individual differences in neuroanatomy or response to the paradigm. Recent studies suggest several parameters, which seem to have an impact on the responsiveness to tES. This could be the individual brain state (81), NMDA receptor configurations (82), growth factors (83) as well as age and education (84).

The calculation of the optimized positioning and weighting of the stimulation electrodes was generalized over the group based on results from earlier studies on unilateral sites. We cannot make any assumptions on effects of a stimulation of the contralateral side. Further, since HD stimulation is more focal, individual differences in head anatomy and the representation of the functional networks are possible and probably more relevant. These could be addressed in future studies using our optimized algorithm on previously obtained imaging data of single subjects, such as fMRI, EEG or simultaneous EEG-fMRI. In any case, making use of imaging techniques will certainly provide more insight in the mechanisms underlying tES. Data could be analyzed in relation to individual theta frequency or phase information of interacting brain regions. These may play an important role for stimulation setups because it can lead to a desynchronization or synchronization and either impair or improve WM performance (42, 69, 85–88).

Although we did only find effects for the high but not for the low load condition, this is not unexpected. Low cognitive demand is obviously related with high performance and can produce ceiling effects. There have been several studies showing tACS-related improvements of WM performance in tasks with higher compared to lower cognitive demand (48, 87, 89).

### 4.6. Conclusion

Overall, the effects of tES are still heterogenous, but HD stimulation regardless of the type of applied current seems to be a feasible method without any significant side effects. Here, we introduce a procedure to calculate an optimal tES model at a given location and orientation inside the head for N stimulation electrodes. Our results suggest, that left frontal theta HD-tACS of the DLPFC is able to improve WM performance, which confirms the involvement of the DLFPC in the visual WM and the relevance of theta oscillations for WM processes. Further investigations involving higher numbers of subjects and a combination with imaging techniques such as EEG and fMRI are needed in order to facilitate tES setups such as individualized stimulation frequencies or locations. Thus, targeting the prefrontal cortex with an optimized HD stimulation setup might be applicable in future clinical studies with patients suffering from neurological or psychiatric diseases with working memory deficits.

## Data availability statement

The raw data supporting the conclusions of this article will be made available by the authors, without undue reservation.

## Ethics statement

The studies involving human participants were reviewed and approved by Ethics Committee of the Medical Association Hamburg. The patients/participants provided their written informed consent to participate in this study.

## Author contributions

JR, CM, and GL contributed to conception and design of the study. JR, MH, SS, and MM collected the data and performed the statistical analysis. JR wrote the first draft of the manuscript. AM and GN wrote sections of the manuscript. All authors contributed to the article and approved the submitted version.

## Funding

This work was supported by the German Research Foundation (SFB 936–178316478 – C6 to CM and GL and Z3 to GN).

## Acknowledgments

The authors would like to thank Till Schneider and Jan Meier for support during the stimulator setup.

## Conflict of interest

The authors declare that the research was conducted in the absence of any commercial or financial relationships that could be construed as a potential conflict of interest.

## Publisher’s note

All claims expressed in this article are solely those of the authors and do not necessarily represent those of their affiliated organizations, or those of the publisher, the editors and the reviewers. Any product that may be evaluated in this article, or claim that may be made by its manufacturer, is not guaranteed or endorsed by the publisher.
